# Patterned Membranes for Proton Exchange Membrane Fuel Cells Working at Low Humidity

**DOI:** 10.3390/polym13121976

**Published:** 2021-06-16

**Authors:** Oliver Fernihough, Holly Cheshire, Jean-Michel Romano, Ahmed Ibrahim, Ahmad El-Kharouf, Shangfeng Du

**Affiliations:** 1 Centre for Fuel Cell and Hydrogen Research, School of Chemical Engineering, University of Birmingham, Birmingham B15 2TT, UK; oxf537@student.bham.ac.uk (O.F.); holly.cheshire@student.bham.ac.uk (H.C.); AXI763@student.bham.ac.uk (A.I.); 2 School of Engineering, University of Birmingham, Birmingham B15 2TT, UK; jean-michel.romano@gadz.org

**Keywords:** PEMFC, proton exchange membrane, catalyst layer, interface, patterned membrane

## Abstract

High performing proton exchange membrane fuel cells (PEMFCs) that can operate at low relative humidity is a continuing technical challenge for PEMFC developers. In this work, micro-patterned membranes are demonstrated at the cathode side by solution casting techniques using stainless steel moulds with laser-imposed periodic surface structures (LIPSS). Three types of patterns, lotus, lines, and sharklet, are investigated for their influence on the PEMFC power performance at varying humidity conditions. The experimental results show that the cathode electrolyte pattern, in all cases, enhances the fuel cell power performance at 100% relative humidity (RH). However, only the sharklet pattern exhibits a significant improvement at 25% RH, where a peak power density of 450 mW cm^−2^ is recorded compared with 150 mW cm^−2^ of the conventional flat membrane. The improvements are explored based on high-frequency resistance, electrochemically active surface area (ECSA), and hydrogen crossover by in situ membrane electrode assembly (MEA) testing.

## 1. Introduction

The extensive commercialisation of proton exchange membrane fuel cells (PEMFCs) is still limited by several technical challenges, which have been identified as performance, cost, and durability [1,2]. Especially, actions are required to reduce the membrane electrode assembly (MEA) cost to meet the DOE targets [3,4]. The membrane’s microstructure can be considered as a fine mesh at either edge with a network of interconnected chambers through its bulk [5]. The membrane allows the transport of protons and water through its structure while inhibiting electron transport and gas permeation [6,7]. The membrane’s water content influences its properties, specifically ionic conductivity. As the membrane hydrates, the acid side chain groups associate with the incoming water molecules through hydrogen bonding [8]. The water improves the transport of protons through the cell, changing the transport mechanism from “Grotthus” to “Vehicular”, as discussed by Elliot et al. [9], and proton-conducting ability increases with the water content. When the membrane is saturated with water, it has the highest conductivity. The only downfall of saturation is that the cathode can become ‘flooded’ with water, which prevents the transport of oxygen to the cathode catalyst layer, reducing the cell’s performance. Water balance in a fuel cell is vital to avoid ‘flooding’ but also to maintain high performance [10].

At each side of the membrane is a catalyst layer, anode and cathode, respectively. An ideal catalyst layer offers a large surface area for the reaction to occur and allows fresh reactants to reach the active site and products to leave without mass transport limitations. Increasing the surface area of reaction is a classic solution to the problem of a slow reaction. This was shown in the development of NASA’s space missions when they reduced the loading of platinum 10-fold from 4 mg cm^−2^ to 0.4 mg cm^−2^ [11] by employing high surface area platinum grains on carbon support (Pt/C). It further reduced when catalyst inks were developed [12]. In a similar vein, 3M produced a nanostructured thin film (NSTF) electrode to increase the electrode active surface area without changing the catalyst formulation [13]. Another recent approach to this is modifying the membrane surface with patterns. In the case of membrane patterning, the idea is that the membrane interface represents the reaction’s surface providing a larger surface area for the catalyst deposition. It is different from making high surface area catalyst materials because this improves the catalyst utilisation ratio by increasing the available triple phase boundary (TPB) within the catalyst layer [14,15,16].

There are three main methods for creating patterned surfaces, namely, casting, imprinting, and ablation. Ablation is possible using high-power X-ray beams; however, due to the destructive nature of the X-ray beam to the membrane bulk, it is not used [17].

Imprinting involves the use of pressure, heat, or both to mould the membrane into the desired surface shape, often done with a silicon or Polydimethlysiloxane (PDMS) master so that it is easy to separate the Nafion from the master after imprinting [16,18,19]. Yildirim et al. [18] used a large feature size of about 20 × 20 micron in a square pattern mould. Their hot-embossed method caused rounding of the features on the membrane in the corners of the mould. They found that the hot pressure treatment of the membrane reduced methanol crossover by a factor of 3, but they cited pattern reproducibility as the main challenge. Bae et al. [16] built on this work using the same configuration of a striped pattern with three different feature sizes: 5/10/1 μm, 5/2/1 μm, and 110/90/70 nm (width/spacing/height, respectively). They found a significant increase in performance for both micro patterns but a drop for the nanopattern. A better pattern replication was achieved by soaking the membranes in water and freely releasing them from the moulds over 24 h. Kim, S. et al. [19] used a specific prism design with a PDMS mould for hot pressing onto Nafion. An increase in power performance was attributed to the pattern pushing water away from the catalyst layer into the gas diffusion layer (GDL).

The casting method, which is employed in this paper, involves using a master mould for casting the ionomer solution to create a membrane with a surface pattern. Several methods have been used to create the master mould, which offers different degrees of pattern replication [14,15]. Zhou et al. demonstrated a 75% improvement in the power density by casting small square features on the flat membrane. They ascribed it to the increase in the cell active area due to the change of the geometric area, and the specific area catalyst loading was lower for the patterned compared with the flat membrane because of 3D topography rather than 2D for flat surface membranes [14]. Jeon et al. [15] tested various patterned features and sizes, namely, circular, square, and hexagonal. These were compared to both a solution cast membrane and a commercial extruded Nafion membrane. They defined the specific area as the increased surface due to 3D features (geometric area) over the cell’s actual area, and these ranged from 1 to 1.698. They showed a power increase of 80% compared with the commercial flat membrane using circular features of 2 µm diameter.

Patterned membranes have been employed in both direct methanol [17,18] and hydrogen [12,13,16] PEMFCs. They have shown performance improvements of up to 80% depending on fabrication technique and catalyst loading [15], attributed to increased catalyst utilisation and greater connection of the platinum to the electrolyte, increasing the TPB homogeneity. Moreover, enhanced water management was reported due to a change in the catalyst layer’s topology, resulting in reduced flooding phenomena [19]. In this work, for the first time, the research is focused on understanding the patterned membrane for application in PEMFCs at low relative humidity (RH). Three different micro patterns, i.e., lotus, lines and sharklet, are applied to the membrane’s cathode side using a one-step solution casting method with a stainless steel master mould. The membranes are then used to fabricate membrane electrode assemblies (MEAs) and evaluated in a single cell to understand their behaviour at different RHs.

## 2. Materials and Methodology

### 2.1. Patterned Membrane Fabrication

The patterns were first created on stainless steel plates by laser-induced periodic surface structures (LIPSS) technique [20] to form the master mould. A controlled laser beam is applied to the stainless steel surface; some of the material is removed, leaving a pattern behind. The patterned stainless steel modules were cleaned using DI water and Isopropyl alcohol (IPA) and used as masters for the solution casting.

The casting process used here was adapted from work reported by Ding et al. [21]. Typically, the required amount of Nafion ionomer dispersion (D1021 10% in water, Ionpower) is measured and mixed with ethanol and water in a sample vile. Here, 3.5 mL of Nafion ionomer dispersion was used for producing a 50 µm membrane thickness [22]. The vile was then placed in a desiccator under vacuum overnight to remove any dissolved gas and prevent bubble formation in the casted membrane. The solution was then poured into a petri dish with the mould placed at the centre. The casted solution was then placed in an oven for 1 h at 100 °C, followed by 120 °C for an additional hour to sinter to create a mechanically robust membrane.

After removing the membranes from the petri dish, the membranes undergo an activation process by immersing in the following solutions at 80 °C for an hour each: DI for cleaning, 3% hydrogen peroxide solution (Sigma Aldrich, Gillingham, UK) to remove any impurities and remaining ionomer, DI water to clean the membrane from hydrogen peroxide, then 0.5 M sulphuric acid (Fisher Chemical, Loughborough, UK) to ensure all the side chains are fully terminated with sulphonic acid groups, and finally in DI water again to remove any excess sulphuric acid.

### 2.2. Membrane Characterisation

#### 2.2.1. Surface Morphology

The surface morphology of the patterned membranes was characterised by SEM analysis performed on a Hitachi TM3030(Tokyo, Japan) plus at 5 kV. Images were taken using the charge-up reduction mode with a BSE detector. Images were taken at the same magnification for comparison of feature sizes and pattern replication. For the cross-sectional analysis, the membrane was freeze broken using liquid nitrogen. The images were taken for the membranes before and after activation and after application of the cathode catalyst layer to observe the change in the pattern feature at each step.

#### 2.2.2. Membrane Swelling and Water Uptake

Swelling and water uptake measurements were performed with a small membrane segment (2 cm^2^). The samples were dried out in an oven at 50 °C overnight. The membrane thickness and weight were measured using a micrometer (RS Pro, Corby, UK) and a microbalance (Sartorius LA120S, Epsom, UK), respectively. The samples were then submerged in DI water and placed in an oven at 50 °C overnight to be fully saturated. The membrane thickness and weight were then measured again. Using the measurements of weight and thickness, the membrane swelling and water uptake were calculated according to Equations (1) and (2).

(1) $$Swelling\;\left(\%\right)=\frac{t_{Wet}-t_{Dry}}{T_{Dry}}\;$$

(2) $$Water\;Uptake\;\left(\%\right)=\frac{m_{Wet}-m_{Dry}}{m_{Dry}}\;$$

where *t* is the thickness and *m* is the mass, measured at both wet and dry conditions.

#### 2.2.3. Ion Exchange Capacity

Ion exchange capacity (IEC) measurements were taken by titration. First, the samples were soaked in water saturated with NaCl for 72 h. Then, the membrane was washed with DI water before titrating 0.1 M NaOH and phenolphthalein until the solution changed its colour. Equation (3) was applied to calculate the IEC [23].

(3) $$\;IEC=\frac{V_{NaOH}C_{NaOH}}{m_{Dry}}$$

where *V* is the volume of NaOH, *C* is the concentration of NaOH, and *m* is the membrane’s dry mass.

### 2.3. MEA Fabrication and In Situ Testing

The patterned membranes were then used to fabricate MEAs with a 5 cm^2^ active area for testing in situ in a single-cell setup. The MEAs were prepared as follows: A 5 cm^2^ gas diffusion electrode (GDE) (Johnson Matthey (London, UK) GDE with 0.4 mg cm^−2^ Pt loading) was used for the anode. A 5 cm^2^ Gas Diffusion Layer (GDL) Sigracet 39BC (SGL Carbon, Wiesbaden, Germany) was used at the cathode, as the cathode catalyst layer was applied directly to the patterned membrane surface. When applying the cathode catalyst layer, the membrane was placed over a target with a 5 cm^2^ area placed on a heated vacuum table at 60 °C. A catalyst ink was prepared to achieve Pt loading of 0.4 mg cm^−2^ by weighing out 10 mg Pt/C catalyst powder (Johnson Matthey, London, UK, HiSPEC 3000 with 20% Pt loading on Carbon) and adding 64 µL of 10% Nafion ionomer dispersant and ethanol and DI water. The ink was then sonicated in a sonication bath for 10 min. The ink solution was then added to an airbrush and sprayed onto the cathode side of the membrane. The anode GDE, the cathode GDL, and the coated membrane were assembled by hot pressing at 130 °C, for 4 min, with 2 of those minutes being under 0.125 tonnes of pressure.

The MEA was then assembled into a single cell and tested using a fuel cell test station (850e, Scribner Associates, VA, USA). Hydrogen and air with controlled temperature and humidity were applied to the anode and cathode side, respectively, and electrochemical measurements including polarisation curve, electrochemical impedance spectroscopy (EIS), and hydrogen crossover were conducted. Moreover, the electrochemical catalyst surface area (ECSA) was measured from the hydrogen adsorption peak obtained via cyclic voltammetry test with the H_2_ and N_2_ environment at the anode and cathode. The EU harmonisation protocol for cell breaking was conducted before performing the measurements [24]. The operating conditions used for testing the MEAs are shown in Table 1.

### 2.4. ECSA Calculation

The data for the ECSA was taken by flowing hydrogen at the anode side and switching the cathode side to pure nitrogen. Then, the potentiostat was used to scan the voltage between 0.1 and 1.2 V. The H^+^ adsorption peak was assessed from the data, using the following equations.

(4) $$ECSA=\frac{Q}{\Gamma }$$

where

(5) $$Q=\int I\;dE\frac{dt}{dE}$$

In Equation (4), *Q* is equal to the total charge under the graph above the double layer. Γ is the charge required to form a monolayer of hydrogen around the surface of the catalyst. *Q* is obtained using equation five, where IdE is the area of the charge obtained and $\frac{dt}{dE}$ is the inverse of the scan rate.

## 3. Results

### 3.1. Patterned Membrane

**Figure 1:**
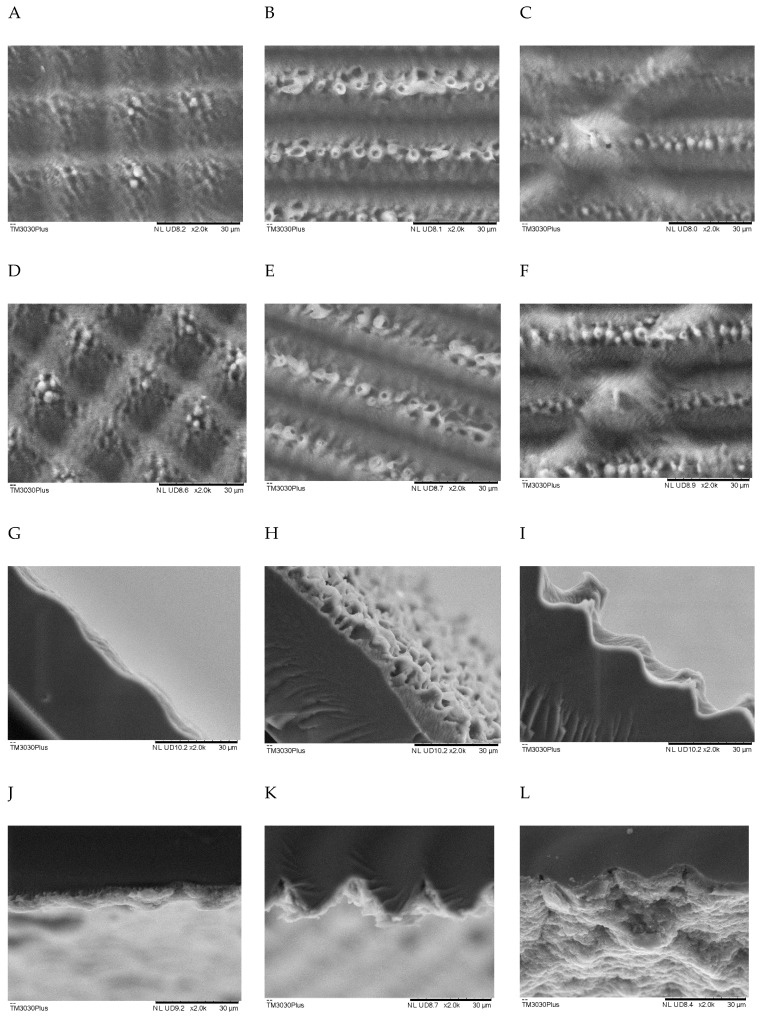
SEM images of the patterned membranes at different fabrication stages; (**A**–**C**) freshly cast, (**D**–**F**) post-activation, (**G**–**I**) cross-section after activation, (**J**–**L**) cross-section after catalyst layer is applied. Lotus pattern (**A**,**D**,**G**,**J**). Lines pattern (**B**,**E**,**H**,**K**). Sharklet pattern (**C**,**F**,**I**,**L**).

The SEM images in Figure 1 show that all patterns exhibit good replication of the master mould from the front view after casting. Minor defects in each pattern are visible in the recesses; this is due to the lasers thermal effect when removing material from the stainless steel mould. The Nafion membrane replicates these features with a high degree of accuracy. Table 2 displays the feature dimensions for the three patterns. The lotus pattern has the shallowest features with a maximum depth of only about 3.5 μm, compared with the 28.1 μm and 16.0 μm of the lines and sharklet patterns. The defects are not as noticeable on this pattern due to the short laser beam scan time (17 min). As expected, the pattern survives activation and is filled up by the application of the catalyst layer. The lines pattern has substantial feature sizes, and the frequency of the defects is greater than the lotus and sharklet patterns. The patterns hold up well to both casting (Figure 1A–C) and activation (Figure 1D–F), and the extent of the defects can be seen in the unsprayed cross-section (Figure 1G–I). The catalyst layer applied conforms to the large features while smoothing some of the defects within its layer (Figure 1J–L). The sharklet pattern has similarly spread defects to the lines pattern, but less intense. The laser protocol is faster to create shallower features for the sharklet (with a depth of only 4.6 μm). It is replicated well by casting and holds up to activation as with the other two. The catalyst layer conforms to the pattern’s shape, with the medium features being visible from the images. The images of the lines pattern catalyst layer show the layer to be less homogeneous than the other patterns. This is to be expected, as the catalyst layer would smooth out the small pattern features and that the larger feature sizes would lead to a less uniform catalyst layer [16,18].

### 3.2. Ion Exchange Capacity, Water Uptake, and Membrane Swelling

**Figure 2:**
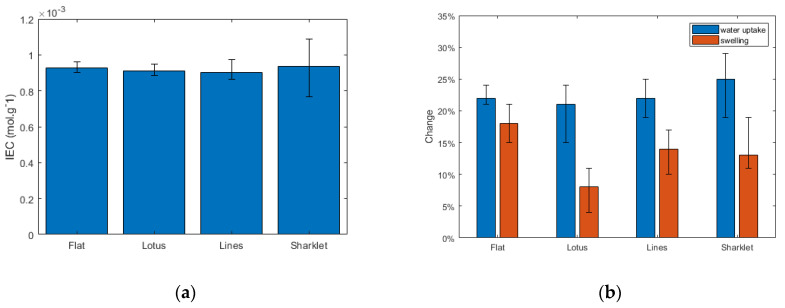
(**a**) Ion exchange capacity, (**b**) water uptake, and swelling ratio for the patterned membranes.

The ion exchange capacity (IEC) was measured to evaluate the influence of the patterns. The results show that the IEC values remain nearly the same (about 0.9 mmol g^−1^) for all membranes despite the surface topography change (Figure 2), which confirms that IEC is a bulk property controlled by the material (Nafion for all samples) and not the surface properties. Similarly, water uptake, again a bulk property, remains similar (21–25%) for all samples. However, the swelling ratio varies with the change of the surface topography. This is believed to be due to the swelling taking place into the pattern gaps and the bulk material showing less bulk dimensional change even though the same amount of water is absorbed. The membrane with the lotus patterns shows the smallest swelling ratio of only about 8%. A low swelling ratio is usually preferred. It reduces the mechanical strain on the membrane with hydration and drying cycles during the fuel cell operation, thus helping to improve the membrane durability [25].

Table 3 gives the membrane thicknesses for all the samples at dry and hydrated conditions; the thicknesses are essential for understanding the relative hydrogen crossover from the patterns.

### 3.3. Membrane Electrode Assembly Test

**Figure 3:**
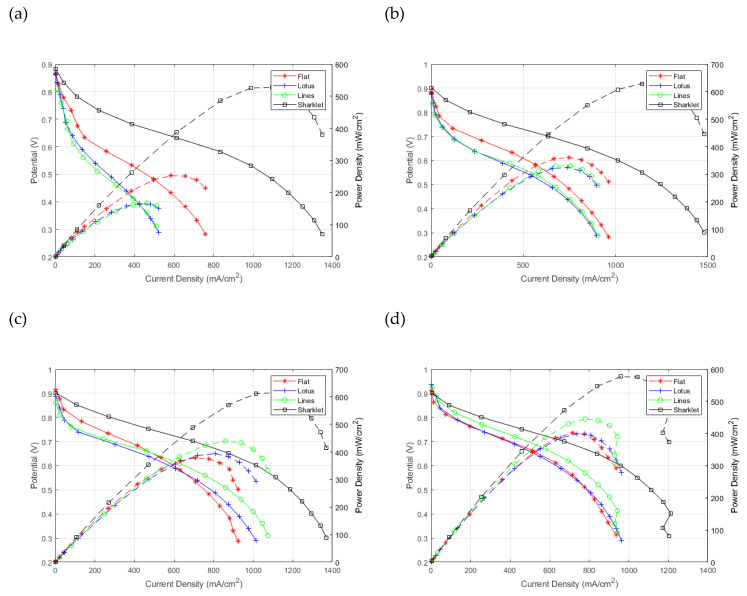
Fuel cell performance for the different membrane patterns at different hydration levels for (**a**) 25%, (**b**) 50%, (**c**) 75%, (**d**) 100%.

The different patterned membranes and a flat sample were used to fabricate MEAs for in situ fuel cell testing. The samples were tested at varied relative humidity conditions, as shown in Figure 3. The sharklet pattern outperformed all other membrane samples at all relative humidity operating conditions. The lotus pattern and flat pattern performed almost identically. There was a deviation in the lotus pattern as relative humidity went to 25%; the similarity was expected because of the minor change to TPB due to the small feature size and depth. However, the low performance of the lotus and lines patterns at 25% RH can be attributed to the larger surface area of the membrane being exposed to the operating conditions, resulting in membrane dehydration.

The lines pattern showed gradual improvement in the performance with the increase in relative humidity. This is probably due to the prominent feature sizes in the pattern resulting in a direct correlation between the reactants’ relative humidity and the hydration of the membrane. Moreover, The TPB area was bigger, making mass transport less hindered at larger current densities. The sharklet pattern showed the best overall performance through all RH values. The most significant improvement was in the mass transport region. However, there is also a shallower ohmic region through the middle of the polarisation curve, meaning the average membrane thickness was smaller and better performing. Better catalyst utilisation explains the changes observed in the sharklet membrane polarisation curve’s activation region despite having the same catalyst loading. The enhanced utilisation is due to a well-connected TPB. This topic is discussed further in the manuscript with the help of the ECSA results.

The drop in performance of the sharklet pattern from 100% to 25% RH is about 20% at 0.6 V; when compared with the flat membrane, this loss is 40%. The lotus shows a loss of 80%, and the lines pattern shows a loss of 85%, again at 0.6 V. The power density of the flat membrane at 0.6V is 0.4 W/cm^2^, compared with the sharklet pattern, which is 0.57 W/cm^2^; an increase of 42%, at 100% RH. Compared with low humidity conditions 25% RH, the flat membrane has a power density of 0.15 W/cm^2^ and the sharklet pattern 0.45 W/cm^2^, a threefold increase measured at 0.6 V.

**Figure 4:**
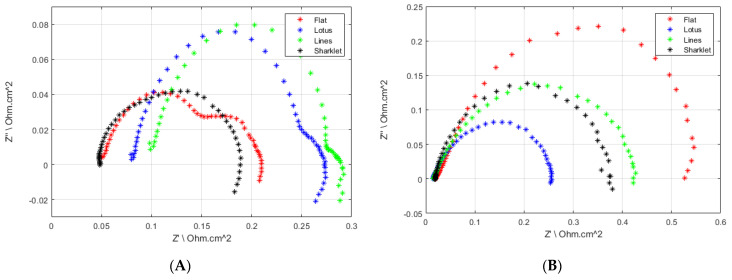
Electrochemical impedance spectroscopy at 0.4 V, 25% (**A**) & 100% (**B**) RH.

Furthermore, EIS analysis was conducted on the MEAs to gain further insight into the performance shown in the polarisation curves. Figure 4A,B shows the EIS results measured at 0.4 V for 25% and 100% relative humidity for the tested samples. The results show the superior properties of the sharklet membrane in terms of low high-frequency resistance (HFR) and mass transport at both relative humidity levels. Generally, HFR increases as RH drops due to the reduction in proton conductivity across the membrane. Le Canut J. et al. [26] showed that shape changes in the impedance spectra could be attributed to drying. All the samples showed a spectral shape change from 100% to 25%, aside from the sharklet pattern, implying it did not dry out as much as the others.

**Figure 5:**
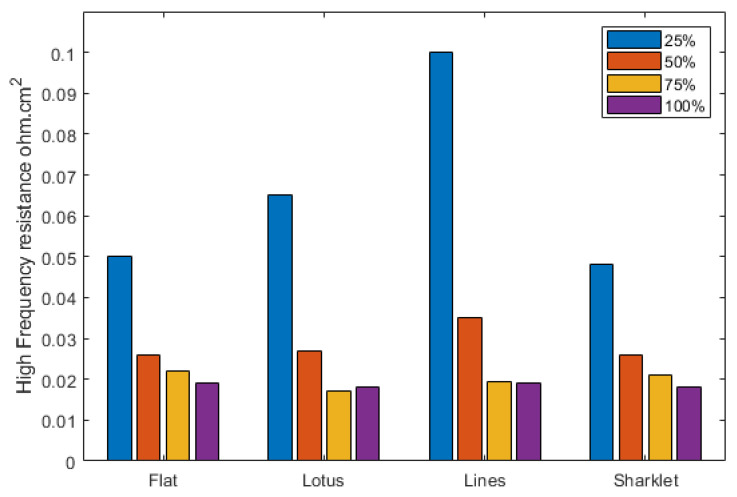
High-frequency resistance across the different humidity levels for each pattern.

HFR is associated with the membrane hydration condition; it has a good correlation with the hydration level and therefore the conductivity of the membrane. Figure 5 shows the HFR variation with humidity for each pattern. The general trend is that the HFR increases as the RH decreases. Figure 5 confirms that the sharklet pattern is least affected by the reactant’s relative humidity conditions, while the lines pattern is the most affected. This correlates well with the polarisation curve results and can be explained by the feature shape and size. The sharklet pattern demonstrates the ability to maintain relatively high proton conductivity at low humidity conditions, allowing the performance to stay high.

**Figure 6:**
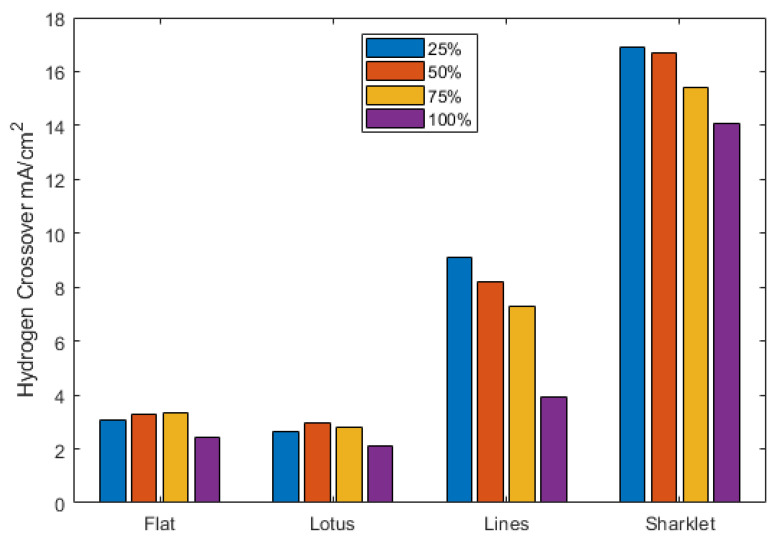
Hydrogen crossover for each pattern at different humidity levels.

Figure 6 shows the crossover current for the different membrane samples at varied relative humidity. Hydrogen crossover appears to slightly vary with relative humidity in the flat and lotus samples, while a significant reduction in the crossover is observed with increasing relative humidity in the lines and sharklet samples. The lotus pattern and flat membrane exhibit very similar behaviour due to the lotus pattern having very little change to the membrane’s bulk thickness. In comparison, the lines and sharklet patterns show a much bigger crossover, especially the sharklet, because it has a considerable pattern depth where the two laser scans superimpose.

The crossover current is much higher for the patterns with the most significant pattern depth [27,28,29]; this is probably due to the local thinning of the membrane at the shallowest parts giving rise to a much shorter diffusion length than a regular flat cast surface. The drop in hydrogen crossover with increasing relative humidity can be explained by membrane swelling and closing the patterns with increasing relative humidity. The sharklet pattern had the highest crossover; however, it did not have the greatest depth. This could be due to some variance in the moulding process, creating a more porous structure; further analysis would be required to confirm this. The crossover explains the relatively lower OCV for the lines and sharklet patterns.

Finally, Table 4 shows the ECSA calculated using the trapezium rule from the corrected H+ adsorption peak. All MEAs were fabricated with a catalyst loading of 0.4 mg cm^−2^. It can be seen that the sharklet pattern has the highest ECSA of all the patterns indicating better catalyst utilisation due to the increase in the TPB area. There was approximately a 10% increase in ECSA from in situ tests relative to the flat membrane MEA. The lower ECSA for the other patterns is probably due to the accumulation of a thick catalyst layer at the surface and in the pattern grooves, resulting in a reduction of catalyst area utilisation. The ECSA results correspond well with the variation in activation losses shown in the polarisation curves, with sharklet demonstrating the smallest and lotus the largest polarisation.

## 4. Discussion

The combination of the right feature size and pattern depth yields the best results; the best performing pattern in this work, the sharklet, did so because of its effects in both the ohmic and mass transport regions, in addition to the increased catalyst utilisation confirmed from the ECSA results (approximately 10% larger than the flat membrane).

The membrane material and thickness govern the ohmic region; the patterning does change the thickness of the membrane, making it thinner in some areas while thicker in others. It also affects the dimensional change when the membrane is hydrated—swelling into the pattern before the bulk thickness is affected. The overall water uptake and ion exchange capacity were unaffected. With a thinner membrane, it is confirmed that the membrane will have a lower ohmic resistance.

Losses in the mass transport region happen when the reactant required at the TPB is more than the supply. This causes a drop in performance to the level that can be supplied. In this case, the patterned surfaces show that it is possible to mitigate against these losses at higher current densities by increasing the TPB surface area. This means that the patterns must be changing the catalyst layer’s transport properties, either allowing more reactant or the removal of products faster than the conventional ones. Both yield the same improvement in performance.

The sharp drop in performance of the lotus and lines patterns, as the RH drops, shows a sharp increase in the slope through the ohmic region. This means that the pattern negatively affected the membrane’s water transport properties, drying it out and increasing its resistance. However, the sharklet pattern showed a stable ohmic region through to 25% RH. This means that the water supplied was effectively redistributed and retained in the membrane keeping the conductivity high. This shows the resilience of the pattern to change in the relative humidity operating conditions in the reactants.

Moreover, the pattern feature size seems to play an important role; the patterns with too shallow features behaved similar to standard membrane surfaces or hinder performance. The large feature size pattern resulted in low tolerance to relative humidity change and increased hydrogen crossover. However, with the optimum feature size and depth demonstrated in the sharklet pattern, the pattern results in a significant improvement in the performance. The performance improvement is more pronounced at a low relative humidity achieving a threefold increase in power density. Furthering this work would involve investigating the sharklet pattern effect, with different feature sizes and relative depth on performance and durability.

## 5. Conclusions

Using steel modified moulds by the LIPSS technique, it is possible to create patterned membranes directly on the steel surface. These membranes showed improved performance across the polarisation curve, especially in the mass transport and ohmic regions. The improvements carried over through the low humidity conditions, which was confirmed by impedance spectroscopy, showing that the high-frequency resistance was reduced. The patterning showed higher catalyst utilisation by an improvement of the ECSA measured in situ. This higher utilisation is probably due to a well-connected TPB as the change in relative feature size showed a drop in performance. The hydrogen crossover was more significant for the patterned compared with the flat membrane.

To build on this work in the future, investigating the feature size effect on performance and hydrogen crossover and assessing the durability of these membranes in long operation scenarios would bring them closer to their effective employment in the next generation of fuel cell cars.

## Figures and Tables

**Table 1:** MEA test conditions in the PEMFC single cell.

Parameters	Anode	Cathode
Fuel	Hydrogen	Air
Temperature (°C)	80	80
Flow rate (mL min^−1^)	150	300
Stoichiometry	1.3	1.5
Relative humidity (%)	25/50/75/100	25/50/75/100
Back pressure (bar)	1.5	1.3

**Table 2:** The feature size dimensions of three patterns.

Pattern	Lotus	Lines	Sharklet
Length (μm)	25	25	25
Diagonal (μm)	5.1	n/a	18.08
Depth (max/average) (μm)	3.5/3.2	28.1	16/4.6

**Table 3:** Average sample thickness, dried and hydrated.

	Dry Thickness μm	Hydrated Thickness μm
Flat	52.33 ± 0.58	62.00 ± 1.00
Lotus	45.00 ± 1.73	48.67 ± 0.58
Lines	53.67 ± 8.96	61.33 ± 11.93
Sharklet	52.00 ± 3.00	58.67 ± 5.69

**Table 4:** ECSA measured from H^+^ adsorption peak in situ.

Pattern	ECSA m^2^ g^−1^
Flat	18.3869
Lotus	12.5417
Lines	17.4702
Sharklet	20.2024

## Data Availability

Data is contained within this article.

## Nomenclature

DOE	department of energy
ECSA	electrochemically active surface area
EIS	electrochemical impedance spectroscopy
GDL	gas diffusion layer
HFR	high frequency resistance
IEC	ion exchange capacity
LIPSS	laser-imposed periodic surface structures
MEA	membrane electrode assembly
NSTF	nano structured thin film
PDMS	polydimethylsiloxane
PEMFC	proton exchange membrane fuel cell
RH	relative humidity
TPB	triple phase boundary

## References

[1] Kirsch D. (2000). The Electric Vehicle and the Budern of History.

[2] Pollet B.G., Kocha S.S., Staffell I. (2019). Current status of automotive fuel cells for sustainable transport. Curr. Opin. Electrochem..

[3] Moreno N.G., Molina M.C., Gervasio D., Robles J.F.P. (2015). Approaches to polymer electrolyte membrane fuel cells (PEMFCs) and their cost. Renew. Sustain. Energy Rev..

[4] (2016). Multi-Year Research, Development, and Demonstration Plan. 3.4 Fuel Cells.

[5] Liang Z., Chen W., Liu J., Wang S., Zhou Z., Li W., Sun G., Xin Q. (2004). FT-IR study of the microstructure of Nafion^®^ membrane. J. Membr. Sci..

[6] Burke K.A. (2003). Fuel Cells for Space Applications.

[7] Faulkner A.J.B.L.R. (2000). Fundamentals and Applications. Annu. Rev. Mater. Sci..

[8] Schalenbach M., Hoefner T., Paciok P., Carmo M., Lueke W., Stolten D. (2015). Gas Permeation through Nafion. Part 1: Measurements. J. Phys. Chem. C.

[9] Elliott J.A., Paddison S.J. (2007). Modelling of morphology and proton transport in PFSA membranes. Phys. Chem. Chem. Phys..

[10] Zawodzinski T.A., DeRouin C., Radzinski S., Sherman R.J., Smith V.T., Springer T.E., Gottesfeld S. (1993). Water Uptake by and Transport Through Nafion^®^ 117 Membranes. J. Electrochem. Soc..

[11] Raistrick I.D. (1989). Electrode Assembly for Use in a Solid Polymer Electrolyte Fuel Cell. U.S. Patent.

[12] Dhar H.P. (1994). Near Ambient, Unhumidified Solid Polymer Fuel Cell. U.S. Patent.

[13] Sinha P.K., Gu W., Kongkanand A., Thompson E. (2011). Performance of Nano Structured Thin Film (NSTF) Electrodes under Partially-Humidified Conditions. J. Electrochem. Soc..

[14] Zhou Z., Dominey R.N., Rolland J.P., Maynor B.W., Pandya A.A., DeSimone J.M. (2006). Molded, High Surface Area Polymer Electrolyte Membranes from Cured Liquid Precursors. J. Am. Chem. Soc..

[15] Jeon Y., Kim D.J., Koh J.K., Ji Y., Kim J.H., Shul Y.-G. (2015). Interface-designed Membranes with Shape-controlled Patterns for High-performance Polymer Electrolyte Membrane Fuel Cells. Sci. Rep..

[16] Bae J.W., Cho Y.-H., Sung Y.-E., Shin K., Jho J.Y. (2012). Performance enhancement of polymer electrolyte membrane fuel cell by employing line-patterned Nafion membrane. J. Ind. Eng. Chem..

[17] Omosebi A., Besser R.S. (2013). Electron beam patterned Nafion membranes for DMFC applications. J. Power Sources.

[18] Yildirim M.H., Braake J.T., Aran H.C., Stamatialis D., Wessling M. (2010). Micro-patterned Nafion membranes for direct methanol fuel cell applications. J. Membr. Sci..

[19] Kim S.M., Kang Y.S., Ahn C.-Y., Jang S., Kim M., Sung Y.-E., Yoo S.J., Choi M. (2016). Prism-patterned Nafion membrane for enhanced water transport in polymer electrolyte membrane fuel cell. J. Power Sources.

[20] Romano J.-M., Garcia-Giron A., Penchev P., Dimov S. (2018). Triangular laser-induced submicron textures for functionalising stainless steel surfaces. Appl. Surf. Sci..

[21] Ding X., Fuller T.F., Harris T.A.L. (2011). Effects of annealing conditions on the performance of solution cast Nafion membranes. ECS Trans..

[22] Branco C.M. (2017). Multilayer Membranes for Intermediate Temperature Polymer Electrolyte Fuel Cells.

[23] Kumar P., Bharti R.P., Kumar V., Kundu P.P. (2018). Progress and Recent Trends in Microbial Fuel Cells.

[24] Tsotridis G., Pilenga A., De Marco G., Malkow T. (2015). EU Harmonised Test Protocols for PEMFC MEA Testing in Single Cell Configuration for Automotive Applications.

[25] Kang J., Kim J. (2010). Membrane electrode assembly degradation by dry/wet gas on a PEM fuel cell. Int. J. Hydrogen Energy.

[26] Le Canut J.-M., Abouatallah R.M., Harrington D.A. (2006). Detection of Membrane Drying, Fuel Cell Flooding, and Anode Catalyst Poisoning on PEMFC Stacks by Electrochemical Impedance Spectroscopy. J. Electrochem. Soc..

[27] Kocha S.S., Yang J.D., Yi J.S. (2006). Characterization of gas crossover and its implications in PEM fuel cells. AIChE J..

[28] Baik K.D., Hong B.K., Kim M.S. (2013). Effects of operating parameters on hydrogen crossover rate through Nafion^®^ membranes in polymer electrolyte membrane fuel cells. Renew. Energy.

[29] Pei P., Wu Z., Li Y., Jia X., Chen D., Huang S. (2018). Improved methods to measure hydrogen crossover current in proton exchange membrane fuel cell. Appl. Energy.

